# Calixarene-mediated assembly of a small antifungal protein

**DOI:** 10.1107/S2052252519000411

**Published:** 2019-02-05

**Authors:** Jimi M. Alex, Martin L. Rennie, Sylvain Engilberge, Gábor Lehoczki, Hajdu Dorottya, Ádám Fizil, Gyula Batta, Peter B. Crowley

**Affiliations:** a School of Chemistry, National University of Ireland, University Road, Galway, Ireland; b Institute of Chemistry, Centre of Arts, Humanities and Sciences, University of Debrecen, Hungary

**Keywords:** conformation selection, molecular glue, nucleating agent, polyethylene glycol, supramolecular chemistry, antifungal proteins, calixarene

## Abstract

Synthetic macrocycles such as calixarenes and cucurbiturils are increasingly applied as mediators of protein assembly and crystallization. The macrocycle can facilitate assembly by providing a surface on which two or more proteins bind simultaneously. This work explores the capacity of the sulfonato-calix[*n*]arene (sclx_*n*_) series to effect crystallization of PAF, a small, cationic antifungal protein. Co-crystallization with sclx_4_, sclx_6_ or sclx_8_ led to high-resolution crystal structures. In the absence of sclx_*n*_, diffraction-quality crystals of PAF were not obtained. Interestingly, all three sclx_*n*_ were bound to a similar patch on PAF. The largest and most flexible variant, sclx_8_, yielded a dimer of PAF. Complex formation was evident in solution via NMR and ITC experiments, showing more pronounced effects with increasing macrocycle size. In agreement with the crystal structure, the ITC data suggested that sclx_8_ acts as a bidentate ligand. The contributions of calixarene size/conformation to protein recognition and assembly are discussed. Finally, it is suggested that the conserved binding site for anionic calixarenes implicates this region of PAF in membrane binding, which is a prerequisite for antifungal activity.

## Abstract 2

PAF, a small cationic antifungal protein has been co-crystallized with a series of anionic calixarenes to reveal novel interaction modes. The largest ligand, sulfonato-calix[8]arene, yielded a PAF dimer in both the solid state and the solution state.

## 1. Introduction   

**Figure 1:**
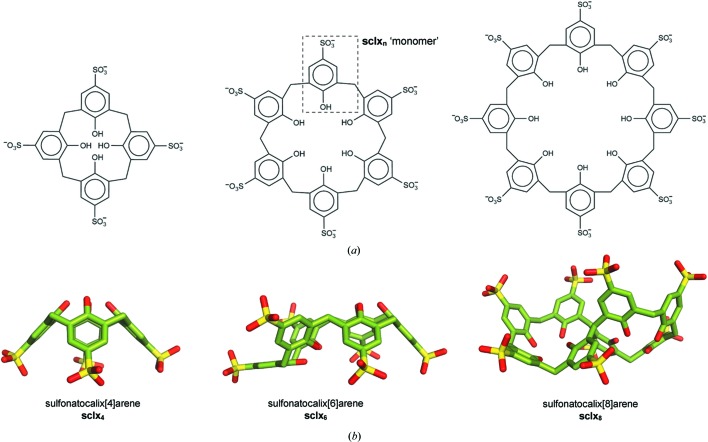
Sulfonato-calix[*n*]arenes. (*a*) Molecular structures and (*b*) cone (sclx_4_), double partial-cone (sclx_6_) and double cone (sclx_8_) conformations.

There is growing interest in the use of synthetic macrocycles as mediators of protein assembly (van Dun *et al.*, 2017 ▸). The special case of protein crystallization (McPherson *et al.*, 2011 ▸) has benefitted from ‘molecular glues’ such as calixarenes and cucurbiturils that promote crystal packing (Guagnini *et al.*, 2018 ▸; Rennie *et al.*, 2018 ▸). The sulfonato-calix[*n*]arenes (sclx_*n*_, Fig. 1 ▸) are highly water-soluble, anionic macrocycles with diverse applications in the biosciences (Baldini *et al.*, 2017 ▸; Giuliani *et al.*, 2015 ▸; Guo & Liu, 2014 ▸). The hydrophobic core and the anionic rim of the calixarene can facilitate protein recognition, in particular, via the entrapment of arginine or lysine side chains (McGovern *et al.*, 2012 ▸, 2014 ▸, 2015 ▸; Wang *et al.*, 2016 ▸; Mallon *et al.*, 2016 ▸; Rennie *et al.*, 2017 ▸, 2018 ▸; Doolan *et al.*, 2018 ▸; Alex *et al.*, 2018 ▸). Consequently, sclx_4_ and related compounds readily co-crystallize with the highly cationic cytochrome *c* and lysozyme (Alex *et al.*, 2018 ▸; Doolan *et al.*, 2018 ▸; McGovern *et al.*, 2012 ▸, 2014 ▸, 2015 ▸). With increasing calixarene size there tends to be more pronounced effects; for example, phosphonato-calix[6]arene (pclx_6_) has an approximately tenfold increase in affinity (with respect to sclx_4_) and prompts dimerization of cytochrome *c* in solution (Rennie *et al.*, 2017 ▸). Sulfonato-calix[8]arene (sclx_8_) on the other hand induces a tetramer of cytochrome *c* (Rennie *et al.*, 2018 ▸). Furthermore, while calix[4]arene is locked in a bowl conformation, the larger calixarenes are flexible and adopt various conformations (Fig. 1 ▸) (Atwood *et al.*, 1992 ▸; Dalgarno *et al.*, 2003 ▸; Gutsche & Bauer, 1985 ▸; Liu *et al.*, 2009 ▸; Perret *et al.*, 2006 ▸; Rennie *et al.*, 2017 ▸, 2018 ▸; Smith *et al.*, 2006 ▸). Accordingly, sclx_8_ can bind to cytochrome *c* either via an extended ‘pleated loop’ or a collapsed ‘double cone’ conformation, as shown using X-ray crystallography (Rennie *et al.*, 2018 ▸).

We were motivated to characterize the sclx_*n*_ series with a single protein and thus investigate systematically how the calixarene size and flexibility influence protein recognition and assembly. Furthermore, we were interested in studying a protein for which a crystal structure was not available. Acknowledging the tendency of sclx_*n*_ to complex cationic proteins we chose the *Penicillium* antifungal protein (PAF) (Marx *et al.*, 1995 ▸, 2008 ▸) as a test case. PAF is a small (∼6.2 kDa, 55 residues) lysine-rich protein (13 × Lys, p*I* ≃ 9) and a potent agent against *Aspergillus* species and dermatophytes (Binder *et al.*, 2010 ▸; Leiter *et al.*, 2005 ▸; Palicz *et al.*, 2016 ▸). The NMR structure is a twisted β-barrel composed of five antiparallel β-strands and stabilized by three disulfide bridges (Batta *et al.*, 2009 ▸; Fizil *et al.*, 2015 ▸, 2018 ▸). Lys30, Phe31, Lys34, Lys35 and Lys38 (loop 3) belong to a conserved region of PAF that is important for antifungal activity (Batta *et al.*, 2009 ▸; Sonderegger *et al.*, 2016 ▸; Garrigues *et al.*, 2017 ▸). Similar to defensins, the mechanism of antifungal action is postulated to require interaction with anionic components on the cell membrane (Binder *et al.*, 2010 ▸; Garrigues *et al.*, 2017 ▸; Silva *et al.*, 2014 ▸). Recent X-ray crystal structures have revealed how defensin–phospholipid binding leads to oligomerization, suggesting a mechanism for membrane permeation (Poon *et al.*, 2014 ▸; Kvansakul *et al.*, 2016 ▸; Cools *et al.*, 2017 ▸; Järvå *et al.*, 2018 ▸). These observations provided further motivation to characterize PAF binding with anionic receptors.

Here, we report three PAF–sclx_*n*_ crystal structures, demonstrating the fitness of calixarenes as crystallization agents. Interestingly, all three calixarenes were bound to PAF, mainly at the conserved loop 3. A similar interaction site was determined by NMR studies; these results suggest that loop 3 is favoured for recognition by anionic receptors. The largest calixarene sclx_8_ mediated a PAF dimer that was observed both crystallographically and in solution. The thermodynamics of PAF–sclx_*n*_ interactions were characterized by isothermal titration calorimetry, providing further evidence of PAF dimerization via sclx_8_. The results are discussed in the context of protein assembly and membrane binding. Finally, insights into protein complexation by flexible calixarenes are provided, including the role of PEG fragments at the protein–calixarene interface.

## 2. Experimental   

### 2.1. Materials   

PAF was produced as described (Batta *et al.*, 2009 ▸; Sonderegger *et al.*, 2016 ▸). The calixarenes were purchased from TCI Chemicals. Stock solutions of sclx_4_, sclx_6_ and sclx_8_ were prepared in water and the pH was adjusted to 6.0.

### 2.2. Crystallization trials   

Co-crystallization experiments were performed by the hanging-drop vapour-diffusion method at 20°C. The reservoir solution was 20–30% PEG 3350 and 50 m*M* sodium acetate, pH 5.6. A range of protein (0.7–7.0 m*M* PAF) and ligand (5–40 m*M* sclx_4_) concentrations were tested for PAF–sclx_4_ co-crystallization. Drops were prepared by combining sequentially 1 µl each of reservoir solution, protein and sclx_4_. Crystals grew at 7 m*M* PAF and 40 m*M* sclx_4_. In the case of PAF–sclx_6_ and PAF–sclx_8_, the protein–ligand solutions were premixed before combining with the reservoir solution. Co-crystals were obtained with 10 m*M* sclx_6_ and 40 m*M* sclx_8_. Crystals grew in 4–5 days (sclx_4_), 2–3 weeks (sclx_6_) or 6–8 weeks (sclx_8_).

The crystallization of ligand-free PAF (7 m*M*) was performed with an Oryx 8 Robot (Douglas Instruments) and a sparse matrix screen (JCSG++, Jena Bioscience). Spherulites were obtained in C6 (40% PEG 300, 100 m*M* potassium phosphate citrate pH 4.2) and needles grew in D7 (40% PEG 400, 100 m*M* Tris–HCl pH 8.5, 200 m*M* lithium sulfate). Manual crystallization trials under these conditions did not yield suitable crystals.

### 2.3. X-ray data collection   

Crystals were cryo-protected in reservoir solution supplemented with 20% glycerol and cryo-cooled in liquid nitrogen. Diffraction data were collected at the SOLEIL synchrotron (France) to 1.30, 1.45 and 1.50 Å for PAF–sclx_4_, PAF–sclx_6_ and PAF–sclx_8_, respectively. Datasets were collected using φ scans of 0.1° over 200° (PAF–sclx_4_), 180° (PAF–sclx_6_) and 110° (PAF–sclx_8_) using an EIGER X 9M detector. In the case of pure PAF, a dataset extending to 3.0 Å was collected for the spherulites (condition C6), but was difficult to index/integrate in both *XDS* and *iMOSFLM*. The needle-like crystals (condition D7) did not diffract.

### 2.4. Structure determination   

The observed reflections for PAF–sclx_4_ were processed with *XDS* (Kabsch, 2010 ▸), whereas *iMOSFLM* (Battye *et al.*, 2011 ▸) was used for the PAF–sclx_6_ and PAF–sclx_8_ datasets. In all cases, the data were scaled using *POINTLESS* (Evans, 2011 ▸) and *AIMLESS* (Evans & Murshudov, 2013 ▸). *Xtriage* (*PHENIX*, Adams *et al.*, 2010 ▸) suggested pseudo-merohedral twinning for the PAF–sclx_4_ data with twin law −*h*, −*k*, −*h* −*l*, and estimated twin fractions of 0.025 (Britton analyses), 0.066 (H-test) and 0.022 (maximum-likelihood method). The structure was determined by molecular replacement in *PHASER* (McCoy *et al.*, 2007 ▸) by using the NMR structure (PDB reference 2mhv, conformer 1; Fizil *et al.*, 2015 ▸) as the search model. A satisfactory solution (LLG, 134; TFZ, 7.4) was obtained with a search model in which residues 1–2, 17–24 and 47–49 were deleted and all six cysteines were replaced by alanine. The coordinates and restraints for sclx_4_ (ligand ID T3Y) were added in *COOT*. Twin refinement did not result in any significant improvement in the electron density. No twinning was indicated for the PAF–sclx_6_ or PAF–sclx_8_ data. The structures were solved by molecular replacement using the structure of PAF–sclx_4_ (devoid of sclx_4_) as the search model. The coordinates for sclx_6_ and sclx_8_ were built in *JLigand* (Lebedev *et al.*, 2012 ▸). High mosaic spread (0.3–0.9) in the PAF–sclx_8_ dataset made it difficult to obtain better *R* values. Truncating the images with high mosaicity did not help in this respect. Iterative cycles of manual model building in *COOT* (Emsley *et al.*, 2010 ▸) and refinement in *BUSTER* (Smart *et al.*, 2012 ▸) were carried out until no further improvements in *R*_free_ and electron density were observed. The final structures were validated with *MolProbity* (Chen *et al.*, 2010 ▸) and deposited in the Protein Data Bank as PAF–sclx_4_ (PDB reference 6ha4), PAF–sclx_6_ (PDB reference 6hah) and PAF–sclx_8_ (PDB reference 6haj).

### 2.5. Accessible surface area calculations   

The effect of sclx_4_, sclx_6_ and sclx_8_ on the accessible surface area (ASA) of PAF residues in the crystal packing environments was determined in *AreaIMol* as described previously (Alex *et al.*, 2018 ▸).

### 2.6. NMR spectroscopy   

The sample conditions were 0.3 or 0.5 m*M* ^15^N-PAF in 10 m*M* sodium phosphate buffer at pH 6.0. NMR titrations were performed at 298 K using 0.5–1 µl aliquots of 50 m*M* stocks of sclx_4_, sclx_6_ or sclx_8_. ^1^H-^15^N HSQC spectra were acquired with spectral widths of 12 p.p.m. (^1^H) and 19 p.p.m. (^15^N) using two scans and 128 increments on a Bruker Avance-II-500 NMR spectrometer. Ligand-induced chemical-shift perturbations were analysed in *CCPN* (Delaglio *et al.*, 1995 ▸).

### 2.7. Isothermal titration calorimetry and data fitting   

PAF samples were dissolved in 10 m*M* sodium phosphate pH 6.0. The same buffer was used to dilute stocks of sclx_4_ (7.1 m*M*, PAF 0.5 m*M*), sclx_6_ (3.6 m*M*, PAF 0.5 m*M*) and sclx_8_ (2.5 m*M*, PAF 0.3 m*M*) to the required concentration. Samples were degassed prior to the titration. Measurements were made at 25°C using a Microcal ITC-200 instrument. Titrations were performed in duplicate with similar trends between each replicate. A single replicate from each calixarene was used for model fitting. Separate titrations of each calixarene into buffer confirmed that the heats of dilution were small, exothermic and approximately constant.

*NITPIC* (Keller *et al.*, 2012 ▸) was used for baseline correction and integration of the thermograms. *Pytc* (Duvvuri *et al.*, 2018 ▸) was used to perform model fitting and parameter estimation. The system of equations relating the independent variables of the model (total concentrations) to the experimental observations (heat generated during injections) for the single-site and bidentate-ligand models are as follows.

Single-site model, 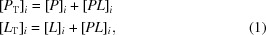


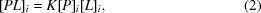


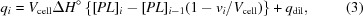
where [*P*_T_]_*i*_ is the total cell concentration of protein at the *i*th injection (independent variable), [*L*_T_]_*i*_ is the total cell concentration of ligand at the *i*th injection (independent variable), *K*_1_ is the equilibrium association constant (fit parameter), Δ*H* is the enthalpy (fit parameter) associated with *K,* *V*_cell_ is the volume of the cell, *v_i_* is the volume of the *i*th injection, *q_i_* is the heat generated from the *i*th injection (dependent variable) and *q*_dil_ is the heat of dilution (fit parameter, assumed to be constant)

Bidentate-ligand model,
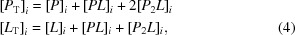


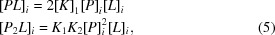


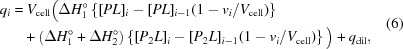
where *K*_1_ and *K*_2_ are the microscopic equilibrium association constants (fit parameters), Δ*H*_1_ and Δ*H*_2_ are the enthalpies (fit parameters) associated with *K*_1_ and *K*_2_, respectively

The expressions for mass balance of the protein and ligand can be represented by equations (1) or (4). Equation (2) or (5) can be used to define the equilibrium constants. For the bidentate ligand model, equation (5) was solved numerically (the Levenberg–Marquardt algorithm) to yield the free-protein ([*P*]*_i_*) and free-ligand ([*L*]*_i_*) concentrations. The free concentrations were used to compute the concentrations of the other states via the equilibrium equations. The heat generated from a given injection was determined using either equations (3) or (6). Parameters were constrained to physically reasonable bounds (*e.g. K*_1_ and *K*_2_ values between 10^2^ and 10^10^ *M* ^−1^) and best-fits were obtained by maximum likelihood starting from a range of initial estimates. Parameter errors and correlations were estimated using a Bayesian approach (Markov chain Monte Carlo simulations). The error for each integrated heat was determined using *NITPIC* (Keller *et al.*, 2012 ▸).

## 3. Results and discussion   

### 3.1. PAF–sclx_*n*_ co-crystallization   

Pure PAF proved to be recalcitrant to crystallization. A sparse-matrix screen yielded spherulites or needle-like crystals only (see experimental[Sec sec2]). In contrast, PAF–sclx_4_ mixtures were crystallized readily from solutions containing PEG and sodium acetate. PAF–sclx_4_, PAF–sclx_6_ and PAF–sclx_8_ co-crystals were obtained at 28–30% PEG 3350 and 50 m*M* sodium acetate pH 5.6 (Fig. S1 and Table S1 of the supporting information).

### 3.2. Data collection and model building   

Datasets extending to 1.30, 1.45 and 1.50 Å resolution were collected from monoclinic (*P*12_1_1) PAF–sclx_4_, PAF–sclx_6_ and hexagonal (*P*6_1_) PAF–sclx_8_ co-crystals, respectively (Table S1). The PAF–sclx_4_ structure was determined using the NMR coordinates (PDB reference 2mhv; Fizil *et al.*, 2015 ▸) as the search model. To obtain a satisfactory solution it was necessary to delete two loops and replace all six cysteines with alanines. After several rounds of model building and refinement a complete PAF structure was obtained. This model was used to solve the PAF–sclx_6_ and PAF–sclx_8_ structures. The PAF fold and the three disulfide bridges in the X-ray structures were consistent with the NMR model (Batta *et al.*, 2009 ▸; Fizil *et al.*, 2015 ▸, 2018 ▸). Interestingly, the fold was altered slightly in response to sclx_*n*_ binding (Fig. S2). Superposition of the three structures revealed a C^α^ r.m.s.d. of 0.54 Å (PAF–sclx_6_) and 0.78 Å (PAF–sclx_8_) relative to PAF–sclx_4_, with the largest differences at loops 2, 3 and 4. The calculated energies of the disulfide bonds (Schmidt *et al.*, 2006 ▸) were approximately threefold lower in the X-ray structures compared with the NMR structure (Table S2).

In contrast to the PAF–sclx_*n*_ crystals, the spherulites and needles of pure PAF failed to provide a usable dataset. The needles did not diffract and the spherulites yielded a 3.0 Å resolution dataset which proved difficult to index and integrate. The difficulty in obtaining suitable crystals of pure PAF suggests that the calixarene facilitates protein assembly and crystallization (Alex *et al.*, 2018 ▸; Doolan *et al.*, 2018 ▸; McGovern *et al.*, 2012 ▸, 2014 ▸, 2015 ▸; Rennie *et al.*, 2017 ▸, 2018 ▸).

### 3.3. Different calixarene, similar binding site   

**Figure 2:**
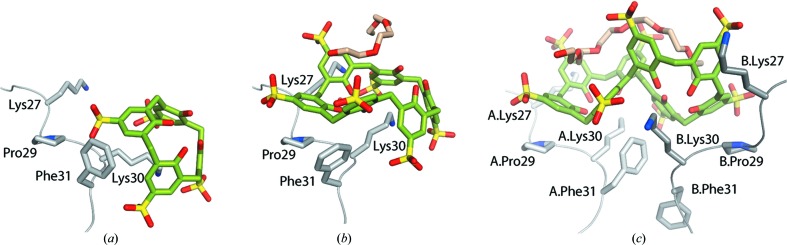
Binding-site interactions in PAF–sclx_*n*_. (*a*) sclx_4_, (*b*) sclx_6_ and (*c*) sclx_8_ binding to PAF at Lys30. Note the altered conformations of Lys30 and Phe31 in each structure, while Pro29 provides a rigid hydrophobic surface for face-to-face interaction with sclx_6_ and sclx_8_. In PAF–sclx_8_, two protein chains interact with the calixarene. PEG fragments equivalent to tetraethylene glycol and heptaethylene glycol were bound to sclx_6_ and sclx_8_, respectively.

The asymmetric unit of the PAF–sclx_*n*_ complexes comprised one (in the case of PAF–sclx_4_ and PAF–sclx_6_) or two (PAF–sclx_8_) molecules of PAF. Each structure contained one calixarene, as shown by the 2*F*_o_—*F*_c_ electron-density maps (Figs. 2 ▸ and S1). Additional electron density adjacent to sclx_6_ and sclx_8_ was modelled as a PEG fragment equivalent to tetraethylene glycol (EG4) and heptaethylene glycol (EG7), respectively (Figs. 2 ▸ and 3 ▸). Sclx_4_, locked in the cone conformation, encapsulates the side chain of a single lysine (Lys30), as observed previously in different protein-clx_4_ complexes (Alex *et al.*, 2018 ▸; Doolan *et al.*, 2018 ▸; McGovern *et al.*, 2012 ▸, 2014 ▸, 2015 ▸). The larger flexible sclx_6_ and sclx_8_ adopted distinct conformations and bound at least two lysines. Sclx_6_ was in the double partial-cone conformation (Atwood *et al.*, 1992 ▸; Dalgarno *et al.*, 2003 ▸), with three sulfonates pointed upwards and three pointed downwards [Figs. 1 ▸(*b*) and 2 ▸(*b*)]. Sclx_8_ adopted the double cone conformation (Liu *et al.*, 2009 ▸; Perret *et al.*, 2006 ▸; Smith *et al.*, 2006 ▸), with each half of the molecule acting like a calix[4]arene to bind one PAF molecule, thus mediating a crystallographic dimer [Fig. 2 ▸(*c*)].

All three calixarenes bound to Lys30, while interacting also with neighbouring residues as well as other proteins (symmetry mates) in the crystal packing. Depending on the ligand size/conformation, the noncovalent contacts varied in their type and multiplicity. The PAF–sclx_4_ complex [Fig. 2 ▸(*a*)] was similar to cytochrome *c*–sclx_4_ (McGovern *et al.*, 2012 ▸), involving a salt bridge and CH—π/cation—π bonds with the encapsulated lysine. Hydrogen bonds to the backbone amide NHs of Lys30, Phe31 and Asp32 were evident and the aromatic ring of Phe31 was in van der Waals contact with an sclx_4_ methylene bridge. Considering symmetry mates [Fig. 4 ▸(*a*)], sclx_4_ formed substantial interfaces (>150 Å^2^) with three proteins. Interestingly, a salt bridge was formed with the N^α^ of Ala1. Salt bridges also occurred with Lys2, Lys17, Lys22 and Lys35, emphasizing a substantial charge–charge component to complexation. In total, the protein–sclx_4_ interfaces buried ∼660 Å^2^ of protein.

Sclx_6_ (1.5 times larger than sclx_4_) also completely encaged Lys30 [Fig. 2 ▸(*b*)]. However, one wall of the calixarene cage was composed of three phenolic groups. The phenolic oxygens were in van der Waals contact with the C^β^, C^γ^ and C^δ^ of Lys30, indicative of CH⋯O hydrogen bonding and the Lys30 N^α^ was hydrogen bonded to a phenolic OH (rather than to a sulfonate). Other differences, with respect to sclx_4_, were water-mediated salt bridges between Lys30 N^ζ^ and two sulfonates and a weak π–π interaction with Phe31 [Fig. 2 ▸(*b*)]. The adjacent residue Pro29 was also important for calixarene binding (*vide infra*). In terms of crystal packing [Fig. 4 ▸(*b*)], the larger sclx_6_ was nestled between five proteins and formed numerous salt bridges (with Lys6, Lys9, Lys11, Lys27, Lys38, Lys42). The resulting protein–ligand contacts mask ∼970 Å^2^ of protein surface. Compared with sclx_4_, the more extensive interactions exhibited by sclx_6_ may explain why four times less ligand was required to achieve crystal growth (see experimental[Sec sec2] and Table S1).

The interactions of sclx_8_ with PAF were similar to those observed with sclx_6_, though less extensive. At twice the size of sclx_4_ it might be expected that sclx_8_ would mask a larger protein surface; however, sclx_8_ formed a PAF dimer [Figs. 2 ▸(*c*) and 4 ▸(*c*)] resulting in a total protein surface coverage of ∼950 Å^2^. The double-cone conformation (compared with the ‘pleated loop’, Rennie *et al.*, 2018 ▸) adopted by sclx_8_ minimized its contact with protein surfaces. Salt-bridge interactions involved up to three lysines from each monomer. Here, again a hydrogen bond was formed between the Lys30 N^α^ and a phenolic OH. In one of the protein chains Phe31 formed an edge-to-face interaction with an sclx_8_ phenolic ring. In protein chain B, Phe31 was disordered [Fig. 2 ▸(*c*)].

In complex with PAF, sclx_4_, sclx_6_ and sclx_8_ contributed an additional surface of ∼550, ∼850 and ∼1290 Å^2^ to the protein, respectively (calculated for a single protein). The exposed calixarene surface is a relatively homogenous ‘mask’ that is conducive to forming noncovalent bridges with other proteins. Apparently, the calixarene acts as molecular glue (Fig. 4 ▸) by providing a patch that mediates protein assembly (subsequently driving protein crystallization) in a special case of the ‘patchy particle model’ (Alex *et al.*, 2018 ▸; Fusco *et al.*, 2014 ▸; James *et al.*, 2015 ▸; Staneva & Frenkel, 2015 ▸; Derewenda & Godzik, 2017 ▸).

**Figure 3:**
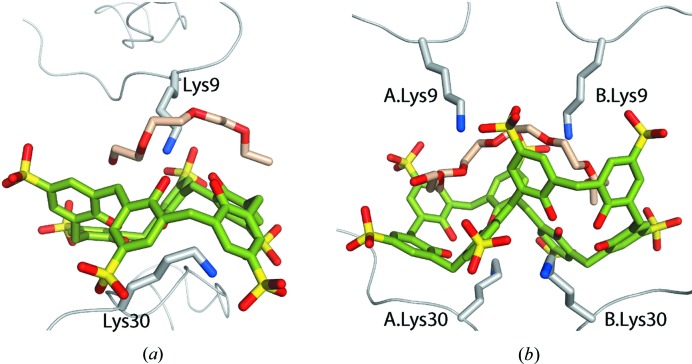
Protein–PEG–calixarene interfaces. The protein–calixarene interfaces are completed by a PEG fragment in (*a*) PAF–sclx_6_ and (*b*) PAF–sclx_8_. Lys9 N^ζ^ simultaneously forms ion–dipole bonds to the PEG (crown-ether-like complex) and a salt bridge to one sulfonate. CH—π and lone-pair—π bonds also occur between PEG and the calixarene phenolic rings.

The presence of PEG fragments (EG4 and EG7) markedly distinguished the PAF–sclx_6_ and PAF–sclx_8_ complexes (Fig. 3 ▸). The PEG–calixarene interaction involved lone-pair–π (Jain *et al.*, 2009 ▸) and CH–π bonds, while the PEG–protein contacts included hydrogen bonds between the oxygen lone pairs and Lys9 (Lys9 N^ζ^⋯O—PEG = 3.0–3.3 Å). This crown-ether like Lys9–PEG interaction resembles the binding of lysine to 18-crown-6 (PDB entry 3wur; Lee *et al.*, 2014 ▸). A heptaethylene glycol fragment has been observed bound to an antibody (PDB entry 2ajs; Zhu *et al.*, 2006 ▸), where it adopted a crown-ether like conformation, compared with the extended conformation in PAF–sclx_8_. In addition, a crystal structure of an SH3 domain (PDB entry 5xg9; Gautam *et al.*, 2017 ▸) revealed various PEG fragments at protein–protein interfaces. These examples suggest that the role of PEG is as an interface ‘filler’ and possibly the PEG fragments (Fig. 3 ▸) contribute towards calixarene conformation selection/stability.

### 3.4. Selectivity of PAF–sclx_*n*_ complexation, why Lys30?   

**Figure 4:**
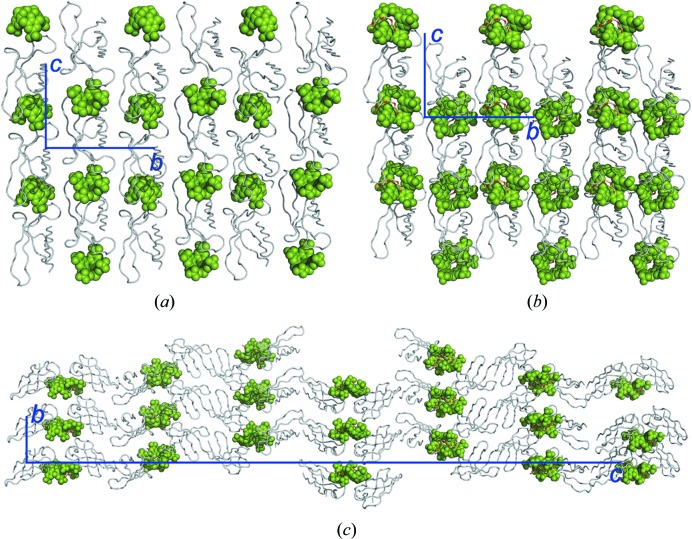
Calixarenes as molecular glues. The crystal packing is dominated by PAF–sclx_*n*_ interactions in (*a*) PAF–sclx_4_, (*b*) PAF–sclx_6_ and (*c*) PAF–sclx_8_. This observation suggests that the calixarene acts as a molecular glue in protein assembly. Proteins, calixarenes and unit-cell axes are depicted in grey, green and blue, respectively. The PEG fragments are depicted as sticks.

**Figure 5:**
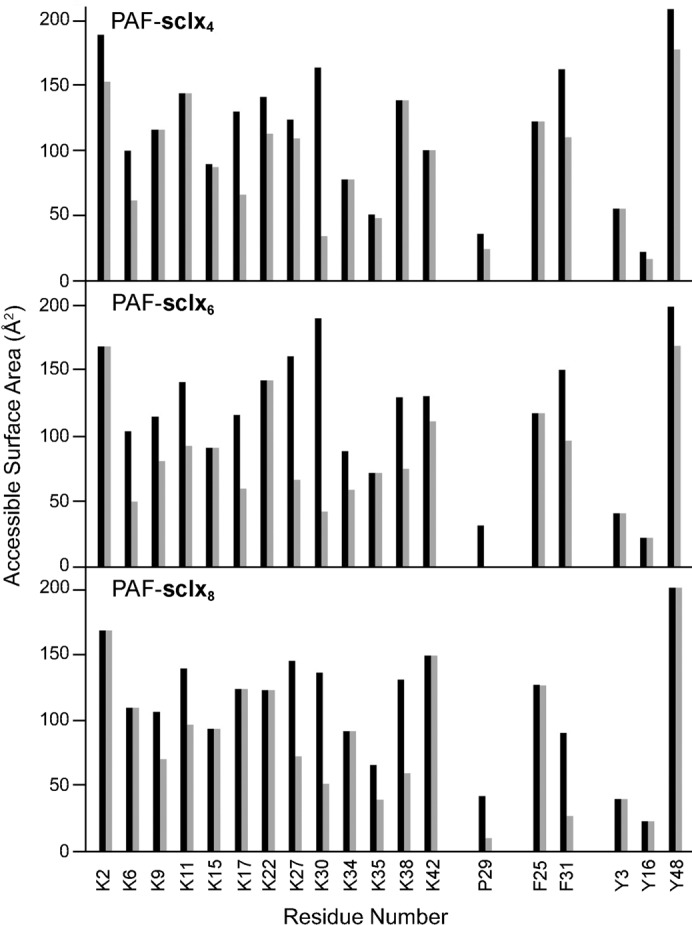
ASA plots. Accessibility of Lys, Pro, Phe and Tyr residues in ligand-free (black) and ligand-bound (grey) PAF. The PAF–sclx_8_ data correspond to chain A.

Considering that PAF contains 13 lysines the question arises as to why Lys30 was selected by sclx_*n*_. ASA calculations were used to probe the selectivity of sclx_*n*_ for the Pro29-Lys30-Phe31 patch over other possible binding sites (Fig. 4 ▸). The calculations accounted for contributions from symmetry mates in the crystal packing (Alex *et al.*, 2018 ▸). The effect of ligand binding on the ASA of all Lys, Pro, Phe and Tyr residues is plotted in Fig. 5 ▸. At least half of the lysines, including Lys30, are highly exposed (ASA ≥ 125 Å^2^) in each structure in the absence of sclx_*n*_. This observation suggests that steric accessibility (McGovern *et al.*, 2014 ▸) was not the determining factor in sclx_*n*_ selectivity. For example, Lys2 (>150 Å^2^) was significantly masked (ΔASA ≥ 15%) by binding with sclx_4_ only. Perhaps a salt-bridge interaction with Asp46 reduced the availability of Lys2 in the other complexes. In contrast, Lys30 was strongly affected by all three calixarenes (ΔASA up to 80%). Adjacent residue Lys27 was also strongly affected in the complexes with sclx_6_ and sclx_8_. The differences in the degree of masking can be attributed to the calixarene sizes (small, sclx_4_) and conformations (‘double cone’, sclx_8_). However, sclx_8_ had more in common with sclx_6_ than sclx_4_. For example, Lys9, Lys11 and Lys38 were 30–50% buried by sclx_6_ or sclx_8_, while sclx_4_ had no effect on these residues. Overall, calixarene binding resulted in significant masking of five (sclx_4_), eight (sclx_6_) and six (sclx_8_) lysines.

PAF has five aromatic residues, Phe25, Phe31, Tyr3, Tyr16 and Tyr48 (Fig. 5 ▸); the latter is highly solvent exposed (∼200 Å^2^) and might be expected to interact with sclx_*n*_. However, only minor contributions were evident (Fig. S3). Phe31 was the dominant aromatic residue for sclx_*n*_ complexation. The adjacent Lys30, Lys34 and Lys35 may facilitate (via charge–charge interactions) calixarene binding here, compared with Tyr48, which is proximal to Lys2 only. The contribution of Pro29 merits special attention as it completes the binding site for both sclx_6_ and sclx_8_ via face-to-face hydrophobic stacks with a phenolic ring [Figs. 2 ▸(*b*) and 2 ▸(*c*)]. These interactions are reminiscent of polyphenol binding to proline-rich proteins (Baxter *et al.*, 1997 ▸; Charlton *et al.*, 2002 ▸; Quideau *et al.*, 2011 ▸). The rigid pyrrolidine ring appears to provide a stable platform for binding the ‘floppy’ sclx_6_ or sclx_8_. Thus, it is perhaps unsurprising that the only proline residue in PAF was involved at the binding site.

As such, it appears to be the combination of the Pro29-Lys30-Phe31 motif and adjacent lysines (charge–charge interactions) that stabilize sclx_*n*_ binding and impart selectivity. This region has been implicated in PAF function, with decreased antifungal activity when Phe31, Lys35 or Lys38 were mutated to Asn or Ala (Batta *et al.*, 2009 ▸; Sonderegger *et al.*, 2016 ▸; Garrigues *et al.*, 2017 ▸). The selectivity of the anionic calixarenes for this site suggests that it may be involved in cell membrane binding and permeation as required for antifungal activity.

### 3.5. NMR characterization and comparison with the solid state   

**Figure 6:**
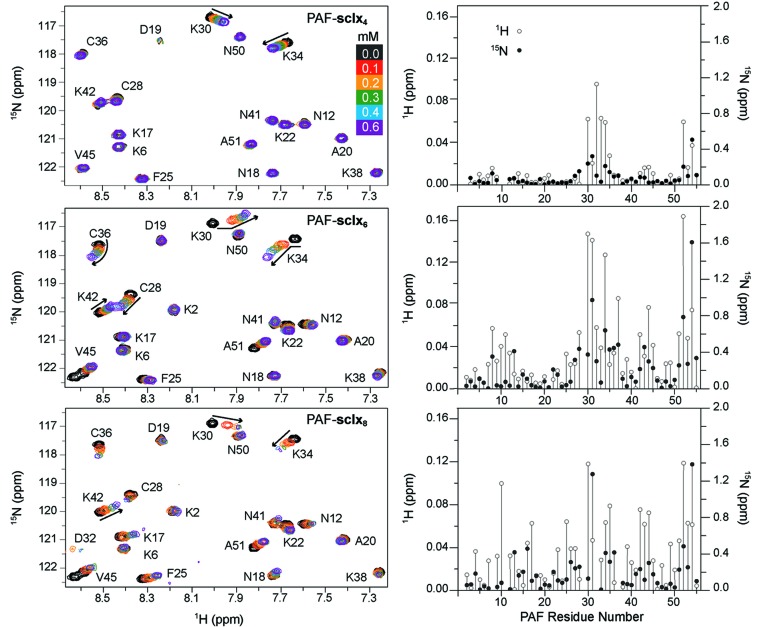
NMR characterization of PAF–sclx_*n*__­_ complexation. (*a*) Region from overlaid ^1^H-^15^N HSQC spectra of pure PAF (black contours) and in the presence of 0.1–0.6 m*M* ligand (coloured scale). Biphasic shifts occurred for resonances Lys30, Lys34 and Cys36 in the presence of sclx_6_. Resonances Lys11, Cys28, Lys30, Lys34 and Cys36 were broadened at 0.3 m*M* sclx_8_, while resonances Thr8, Lys11, Asp32 and Thr37 were broadened beyond detection at 0.6 m*M* sclx_8_. (*b*) Plots of chemical-shift perturbations measured for PAF backbone amides in the presence of 0.6 m*M* sclx_4_, sclx_6_ or sclx_8_. Blanks correspond to Pro30 and undetectable resonances (due to broadening).

PAF–calixarene binding in solution was assessed by NMR spectroscopy. Titrations were performed by the addition of microlitre aliquots of sclx_*n*_ to ^15^N-labelled PAF, which was monitored by ^1^H-^15^N HSQC spectroscopy (Fizil *et al.*, 2018 ▸; McGovern *et al.*, 2012 ▸). The overlaid spectra (Fig. 6 ▸) revealed increasing chemical-shift perturbations (Δδ) as a function of sclx_4_ or sclx_6_ concentration, indicative of fast to intermediate exchange between the ligand-free and ligand-bound states. Some biphasic shifts were evident for sclx_6_. Severe broadening effects were observed with ≥0.3 eq sclx_8_, indicative of a slow-exchange process and suggesting the possibility of ligand-mediated oligomerization (Doolan *et al.*, 2018 ▸; Fonseca-Ornelas *et al.*, 2017 ▸; Mallon *et al.*, 2016 ▸; Rennie *et al.*, 2017 ▸, 2018 ▸).

The Δδ plot (Fig. 6 ▸) shows a clear selectivity for sclx_4_ binding to Lys30 and neighbouring residues 31–36. In the crystal structure, all of these residues occurred in the vicinity of sclx_4_. Significant Δδ were observed also for the C-terminal Val52 and Cys54, which are further from the crystallographic binding site. However, both of these residues are adjacent to Pro29, and Cys54 is hydrogen bonded to Lys34, suggesting a mechanism for how these resonances sense ligand binding. In the presence of sclx_6_, the Δδ plot again shows a preference for binding around Lys30 as well as effects at the C-terminus (Val52 N^α^ is hydrogen bonded to sclx_6_). However, compared with sclx_4_, the shifts are 2–4 times larger and other segments of the primary structure (residues 6–13 and 42–45) were also affected. These two regions correspond to additional sclx_6_ binding sites evident in the crystal packing. Therefore, the NMR data suggests that the PAF–sclx_6_ interaction fluctuates, with the calixarene exploring different patches on the protein surface, as observed previously for cytochrome *c*–sclx_4_ complexes (Doolan *et al.*, 2018 ▸; McGovern *et al.*, 2012 ▸). Judging from the magnitude of the shifts, binding to Lys30 is preferred while a weaker interaction occurred at a patch involving Lys6 and Lys42.

The titrations with sclx_8_ resulted in different effects. In addition to pronounced perturbations of Lys30 and neighbours, substantial broadening effects occurred. Cys28, Lys30, Lys34 and Cys36 broadened at 0.3 m*M*, and Thr8, Lys11, Asp32 and Thr37 broadened beyond detection at 0.6 m*M* sclx_8_. These eight residues are located at the crystallographically defined binding site. Thus, the broadening effects may be indicative of PAF dimerization, consistent with the sclx_8_-mediated dimer in the crystal structure [Fig. 2 ▸(*c*)]. Previously, we observed a complete loss of the HSQC spectrum of cytochrome *c* in complex with pclx_6_, which also yielded a dimer in the solid state (Rennie *et al.*, 2017 ▸).

### 3.6. Thermodynamics of PAF–sclx_*n*_ complexation   

**Figure 7:**
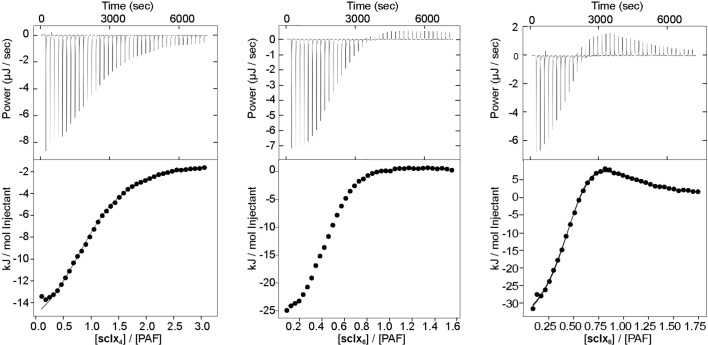
ITC analysis of PAF–sclx_*n*_ complexation. Top panels show the baseline-corrected thermograms for injections of sclx_4_, sclx_6_ or sclx_8_ into PAF. Bottom panels are the observed heats (data points) and the fits (solid line) for single-site (sclx_4_) and bidentate-ligand (sclx_8_) models.

Isothermal titration calorimetry was used to characterize the PAF–sclx_*n*_ binding affinities and stoichiometries (Fig. 7 ▸). The data were fitted to a single-site or a bidentate-ligand model. The latter model describes a bidentate ligand that can bind two protein molecules and was necessary to describe the obviously biphasic data for sclx_8_. The choice of this model is supported by the observation of a PAF–sclx_8_–PAF dimer in the crystal structure, and by the spectral broadening in the NMR experiments. All of the fit parameters were well determined by the data (Table 1 ▸), with parameter errors assessed by Bayesian methods (Patil *et al.*, 2010 ▸).

The isotherms for sclx_4_ injected into PAF were fitted to a single-site binding model with *K*_d_ ∼110 µ*M*. In contrast, the isotherms for sclx_8_ were biphasic (Brautigam, 2015 ▸) and fitted to a bidentate ligand model with *K*_d_ values of ∼10 and ∼30 µ*M*, for binding the first and second molecule of PAF, respectively. The isotherms for sclx_6_ were intermediate between sclx_4_ and sclx_8_, suggesting that this ligand may exhibit weak bidentate binding. A satisfactory fit for this data was not obtained with either model. The ITC data demonstrate an increasing affinity for PAF as the calixarene size increases and a switch in binding mode from the small, rigid sclx_4_ (single site) to the large, flexible sclx_8_ (bidentate).

## 4. Conclusions   

Using a combination of X-ray crystallography and NMR spectroscopy it was demonstrated that the sclx_*n*_ series binds selectively to the highly cationic PAF. Despite the varying size and conformational flexibility, sclx_4_, sclx_6_ and sclx_8_ bound similarly the Pro29-Lys30-Phe31 motif in loop 3. The selectivity of the anionic calixarenes for this motif, and the role of loop 3 in antifungal activity, suggests that this region may be required for membrane binding. In addition to charge–charge interactions (showed by numerous lysine-to-sulfonate salt bridges), other noncovalent bonds including CH–π and π–π (via Pro29 and Phe31, respectively) participated in ligand stabilization. The presence of PEG fragments at the protein–sclx_6_ and protein–sclx_8_ interfaces suggests that PEG acts as a ‘filler’ to complete the binding site, potentially reinforcing the calixarene conformation.

The structures of all three PAF–sclx_*n*_ co-crystals highlight the potential of calixarenes as a ‘sticky patch’ on the protein surface that facilitates assembly and crystallization. In the case of the sclx_4_ and sclx_6_ co-crystals (*P*12_1_1), it is evident that the calixarene is a dominant contributor to the crystal packing (Fig. 4 ▸). Similarly in the sclx_8_ structure (*P*6_1_), the packing involves substantial protein–calixarene contacts, and the structure is interesting as sclx_8_ mediates a PAF dimer. Previously, we found that sclx_8_ mediates a tetramer of cytochrome *c* (Rennie *et al.*, 2018 ▸). Generally, it seems that calixarene-mediated protein crystallization may be a special case of the patchy particle model for protein assembly (Alex *et al.*, 2018 ▸; Fusco *et al.*, 2014 ▸; James *et al.*, 2015 ▸; Staneva & Frenkel, 2015 ▸; Derewenda & Godzik, 2017 ▸). Considering that PAF alone did not yield diffraction-quality crystals, we conclude that co-crystallization with sclx_*n*_ was beneficial. Anionic calixarenes may generally facilitate crystallization and structure determination of small cationic proteins.

The binding surfaces observed in the NMR experiments were consistent with the X-ray data. However, the NMR effects were more pronounced with increasing calixarene size, suggesting that the larger calixarenes mask a greater portion of the protein surface and/or lead to assembly in solution. Similarly, the ITC experiments revealed tighter affinities and more complex effects with increasing calixarene size. In particular, sclx_8_ behaved as a bidentate ligand that facilitated PAF dimerization. These data add to the growing evidence of calixarene-mediated protein assembly in solution (Doolan *et al.*, 2018 ▸; Rennie *et al.*, 2017 ▸, 2018 ▸). In terms of the biological relevance of these data it is noted that defensin oligomerization (upon phospholipid binding) has implications for antifungal activity (Poon *et al.*, 2014 ▸; Järvå *et al.*, 2018 ▸). Perhaps calixarenes can be used to modulate the activity of PAF and related proteins.

## Supplementary Material

Supporting information. DOI: 10.1107/S2052252519000411/lq5018sup1.pdf

PDB reference: PAF–sclx4 co-crystal, 6ha4

PDB reference: PAF–sclx6 co-crystal, 6hah

PDB reference: PAF–sclx8 co-crystal, 6haj

## Figures and Tables

**Table 1:** Thermodynamics of PAF–sclx_*n*_ complexation determined by ITC Fit values are median (2.5% quantile, 97.5% quantile) from the Markov chain Monte Carlo method. In the case of sclx_6_, the fit parameters for both models are shown.

[Ligand]† (µM)	[PAF]‡ (µ*M*)	*K*_d_ (µ*M*)	Δ*H* (kJ mol^−1^)	*T*Δ*S* (kJ mol^−1^)
PAF–sclx_4_ (single-site model)				
7143 (1248)	500 (412)	107.0 (0.0, 0.0)	−16.9 (0.1, 0.1)	−5.6 (0.2, 0.2)
				
PAF–sclx_6_ (single-site model)				
3623 (633)	500 (412)	15.4 (0.0, 0.0)	−28.2 (0.2, 0.1)	−0.7 (0.2, 0.2)
				
PAF–sclx_6_ (bidentate-ligand model)				
3623 (633)	500 (412)	47.8 (0.0, 0.0)	−9.2 (0.1, 0.2)	−15.4 (0.4, 0.3)
		45.8 (3.5, 4.1)	−20.1 (0.4, 0.4)	−4.6 (0.6, 0.6)
				
PAF–sclx_8_ (bidentate-ligand model)				
2500 (437)	300 (247)	10.6 (1.3, 1.4)	−3.5 (0.3, 0.3)	−24.8 (0.3, 0.3)
		33.5 (4.8, 6.5)	−36.0 (1.7, 1.4)	10.5 (1.8, 2.1)

† Calixarene concentrations in the syringe; the final concentrations are indicated in parentheses.

‡ Protein concentrations in the cell; the final concentrations are indicated in parentheses.
