# Supplementary material for: Calixarene-mediated assembly of a small antifungal protein
Source: IUCrJ. 2019 Feb 5;6(Pt 2):238–47. doi: 10.1107/S2052252519000411 (PMC6400181; doi:10.1107/S2052252519000411)
Supplement: Supplementary file 1 [file m-06-00238-sup1.pdf]

IUCrJ

Volume 6 (2019)

Supporting information for article:

Calixarene-mediated assembly of a small antifungal protein

Jimi M. Alex, Martin L. Rennie, Sylvain Engilberge, Gábor Lehoczki, Hajdu Dorottya, Ádám Fizil, Gyula Batta and Peter B. Crowley

Table S1. X-ray data collection, processing and refinement statistics for PAF-$sclx_n$ complexes

		Crystallization	
	**PAF-$sclx_4$**	**PAF-$sclx_6$**	**PAF-$sclx_8$**
**[PAF] / [$sclx_n$] (mM)**	7 / 40	7 / 10	7 / 40
**PEG 3350 (%)**	30	30	28
**Buffer**		0.05 M sodium acetate pH 5.6	
		Data Collection	
**Light source**		Soleil, PROXIMA 2A	
**Wavelength (Å)**		0.980105	
**Space group**	$P12_11$	$P12_11$	$P6_1$
**Cell constants**	a = 22.71 Å<br>b = 37.58 Å<br>c = 29.95 Å<br>$\alpha = \gamma = 90°$<br>$\beta = 111°$	a = 24.74 Å<br>b = 38.59 Å<br>c = 29.93 Å<br>$\alpha = \gamma = 90°$<br>$\beta = 112°$	a = 24.30 Å<br>b = 24.30 Å<br>c = 313.69 Å<br>$\alpha = \beta = 90°$<br>$\gamma = 120°$
**Resolution (Å)**	27.82-1.33<br>(1.37-1.33)	27.08-1.45<br>(1.48-1.45)	21.06-1.50<br>(1.60-1.50)
**# unique reflections**	10142 (842)	8592 (397)	16146 (2346)
**Multiplicity**	3.4 (2.9)	2.9 (2.5)	4.8 (4.0)
**I/σ (I)**	6.1 (1.3)	11.5 (5.1)	8.4 (1.0)
**Completeness (%)**	92.2 (75.7)	94.7 (90.3)	96.8(97.7)
**R_{meas}[b] (%)**	12.1 (61.1)	7.4 (33.9)	8.9 (98.2)
**R_{pim}[c] (%)**	6.2 (34.2)	4.2 (20.4)	3.7 (47.4)
**$CC_{1/2}$**	0.986 (0.742)	0.992 (0.760)	0.998 (0.226)
**Solvent content (%)**	35	41	43
		Refinement	
**R_{work}**	0.185	0.200	0.217
**R_{free}**	0.217	0.236	0.244
**rmsd bonds (Å)**	0.009	0.011	0.011
**rmsd angles (°)**	1.250	1.41	1.450
**# molecules in asymmetric unit**			
**Protein**	1	1	2
**Ligand**	1	1	1
**Water**	55	57	77
**Ave. B-factor (Å^2)**	20.82	23.86	30.01
**Ramachandran analysis[d]**<br>**% residues in**			
**favoured regions**	100.0	100.0	95.3
**allowed regions**			3.76
**PDB code**	6ha4	6hah	6haj

[a]Values in parentheses correspond to the highest resolution shell [b]$R_{meas} = \sum_{hkl} \sqrt{(n/n-1)} \sum_{l} |I_i(hkl) - \langle I(hkl) \rangle| / \sum_{hkl} \sum_i I_i(hkl)$; [c]$R_{pim} = \sum_{hkl} \sqrt{(1/n-1)} \sum^{n}_{i=1} |I_i(hkl) - \langle I(hkl) \rangle| / \sum_{hkl} \sum_i I_i(hkl)$; [d]Calculated in MolProbity.

Table S2. Calculated energies of the disulfide bonds in PAF.

**Structure**[a]	**Torsion**[b]					**Energy**[c] **(kJ/mol)**
	χ_1	χ_2	χ_3	$\chi_{1'}$	$\chi_{2'}$	
PAF-**sclx$_4$**	176.6	68.6	79.6	119.4	55.5	8.9 ± 3.1
X-ray, 6HA4	67.9	89.1	77.4	87.1	-168.2	
	-54.0	-90.9	-87.4	176.9	-53.8	
PAF-**sclx$_6$**	178.2	65.7	83.1	117.8	50.0	7.8 ± 3.3
X-ray, 6HAH	67.5	89.5	77.7	82.0	-172.1	
	-60.3	-81.9	-86.1	168.6	-52.5	
PAF-**sclx$_8$**	178.5	70.5	83.1	112.9	49.4	7.9 ± 3.0
X-ray, 6HAJ	63.1	90.3	78.8	82.6	-172.0	
	-56.3	-83.9	-90.2	173.7	-49.3	
PAF	-154.9	-172.1	-98.1	-112.7	72.7	27.7 ± 9.3
NMR, 2MHV	82.1	126.6	-133.0	-95.3	-59.3	
	-102.3	-149.7	96.7	118.4	-151.6	

[a]The structure composition, type and PDB id are indicated
[b]Five torsion angles for each of 3 disulfide pairs (Cys7-Cys36, Cys14-Cys43, Cys28-Cys54).
[c]The calculated energy averaged over the three disulfide bonds.

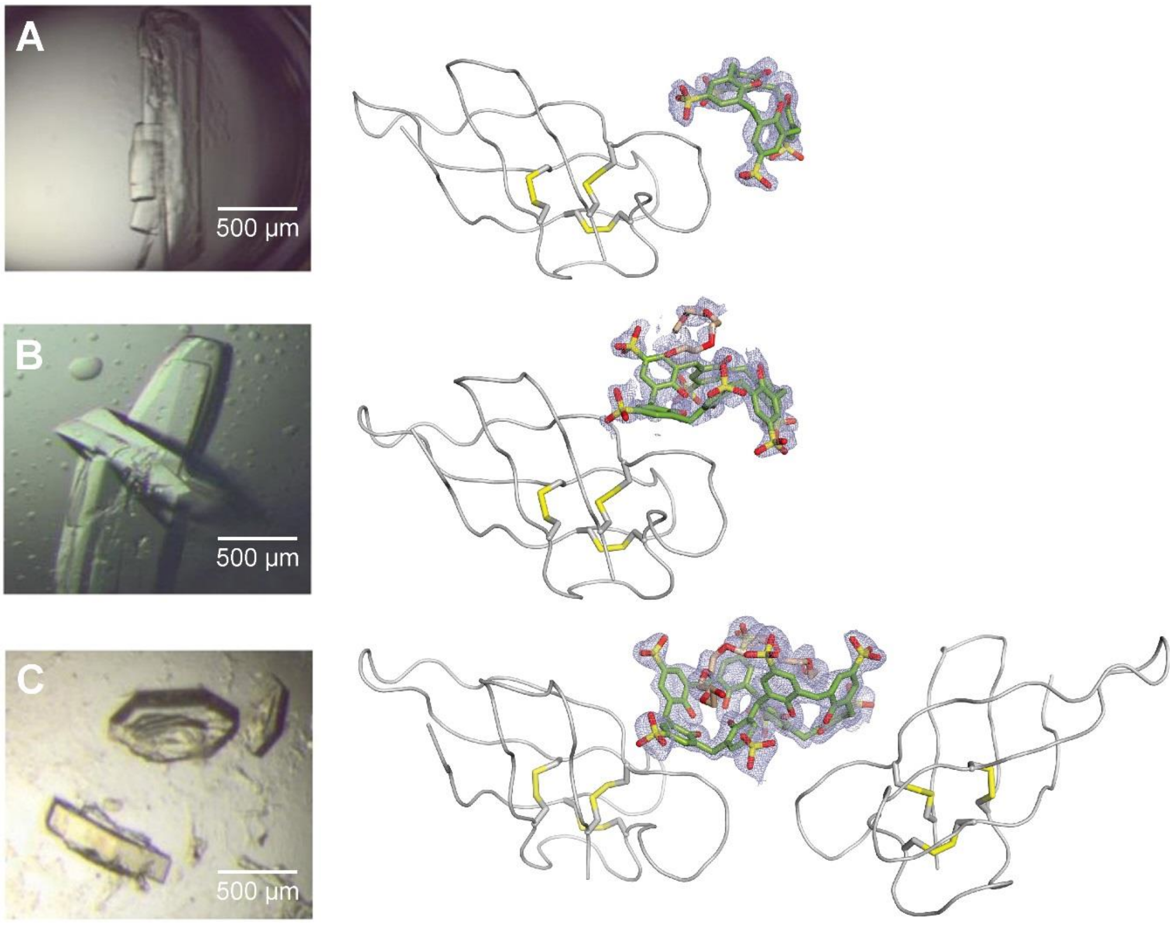

Figure S1. PAF-sclx$_n$ crystals and X-ray structures. Diffraction-quality co-crystals and the asymmetric units of PAF with **(A) sclx$_4$ (B) sclx$_6$** and **(C) sclx$_8$**. The asymmetric unit comprised one protein and one ligand (A,B) or two proteins and one ligand (C). The unbiased $2F_o - F_c$ electron density map (contoured at 1.0 σ) showing calixarenes, and PEG fragments in (**B** and **C**).

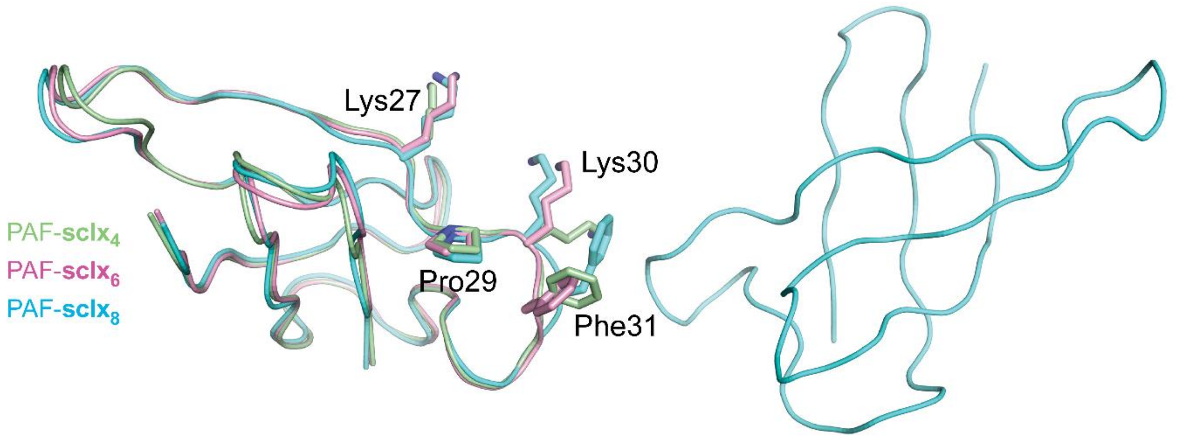

Figure S2. PAF backbone flexibility and binding site conformations. Superposition of the three crystal structures highlight changes in the PAF backbone. The side chains of Lys27, Lys30 and Phe31 have different conformations, while Pro29 provides a rigid platform for hydrophobic interactions with **sclx$_6$** and **sclx$_8$**. The calixarene coordinates are not shown.

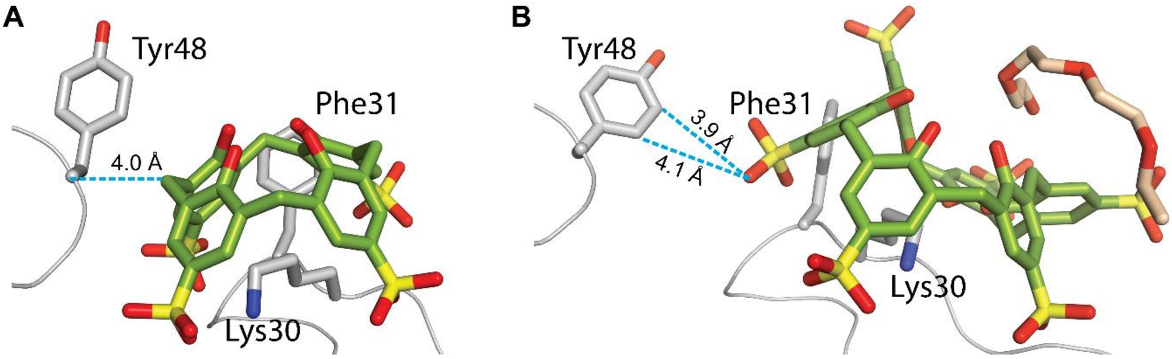

Figure S3. Tyr48-calixarene Interactions. (A) Tyr48-C$^{\alpha}$ in van der Waals contact with a methylene bridge of **sclx$_4$**, **(B)** Tyr48-C$^{\delta/\varepsilon}$ form weak anion-quadrupole bonds with a sulfonate of **sclx$_6$**.
